# Use of ivabradine and atorvastatin in emergent orthopedic lower limb surgery and computed tomography coronary plaque imaging and novel biomarkers of cardiovascular stress and lipid metabolism for the study and prevention of perioperative myocardial infarction: study protocol for a randomized controlled trial

**DOI:** 10.1186/1745-6215-15-352

**Published:** 2014-09-07

**Authors:** Nima Rudd, Ivan Subiakto, Muhammad Asrar ul Haq, Vivek Mutha, William J Van Gaal

**Affiliations:** Department of Cardiology, The Northern Hospital, 185 Cooper Street, Epping, 3076 VIC Australia; Department of Medicine, University of Melbourne, Grattan St, Melbourne, 2010 VIC Australia

**Keywords:** Ivabradine, Perioperative myocardial infarction, Atorvastatin, Computed tomography coronary plaque, Novel biomarkers, Lipid metabolism, Postoperative arrhythmia, Coronary artery disease

## Abstract

**Background:**

The incidence of perioperative myocardial infarction (PMI) globally is known to be around 2 to 3% and can prolong hospitalization, increased morbidity and mortality. Little is known about the pathophysiology and risk factors for PMI. We investigate the presence of elevated novel cardiac markers and preoperative coronary artery plaque through contemporary laboratory techniques to determine the correlation with PMI, as well as studying ivabradine and atorvastatin as protective pharmacotherapies against PMI in the context of orthopedic surgery.

**Methods/Design:**

We aim to enroll 200 patients aged above 60 years who suffer from neck of femur fracture requiring surgery. Patients will be randomized to four arms (no study drugs, atorvastatin only, ivabradine only and ivabradine and atorvastatin). Our primary outcome is incidence of PMI. All patients will receive an electrocardiogram, cardiac echocardiography, measurement of novel cardiac biomarkers and computed tomography (CT) coronary angiography. A telephone interview post discharge will be conducted at 30 days, 60 days and 1 year.

**Discussion:**

We postulate that ivabradine and atorvastatin will reduce the rate and magnitude of PMI following surgery by reducing heart rate and attenuating catecholamine-induced tachycardia postoperatively. Secondly, we postulate that postoperative reduction in heart rate and catecholamine-induced tachycardia with ivabradine will correlate with a reduction in cardiovascular novel biomarkers which will reduce atrial stretch and postoperative incidence of arrhythmia. We aim to demonstrate that treatment with ivabradine and atorvastatin will cause a reduction in the incidence and magnitude of PMI, the benefit of which is derived primarily in patients with greater atherosclerotic burden as measured by higher CT coronary calcium scores.

**Trial registration:**

This study protocol has been listed in the Australia New Zealand Clinical Trial Registry (registration number: ACTRN12612000340831) on 23 March 2012.

## Background

In the United States approximately 24 million surgical procedures are performed annually [1], and it is estimated that 1 million of these operations will be complicated by a perioperative cardiovascular event [2]. With more than 230 million major surgical operations being performed every year worldwide, the scope of this problem is massive [3].

Among patients undergoing major non-cardiac surgery, the overall incidence of perioperative myocardial infarction (PMI) is 2 to 3%, and in higher risk populations, such as those patients undergoing vascular surgery, rates can be as high as 34% globally [4, 5]. PMI is clinically a very significant occurrence, associated with prolonged hospital stay, substantial morbidity and mortality rates as high as 25 to 40% [6, 7].

The definition of PMI is based on the recent universal definition and requires a rise and fall of cardiac biomarkers (preferably troponin), with at least one measurement above the 99th percentile of the upper reference limit, in the setting of either symptoms of ischemia, electrocardiogram (ECG) changes or imaging evidence of necrosis [8]. However, the majority of postoperative troponin elevations are asymptomatic, without ECG or imaging evidence of infarction, yet still confer a substantially increased risk of death [9–11]. As such, the distinction between infarction and necrosis is often subtle, and many patients will not meet the criteria for infarction simply based on the absence of routine postoperative cardiac imaging. Furthermore, small myocardial infarcts usually do not produce wall motion abnormalities on an echocardiogram., As such, imaging evidence of small infarcts will rely on cardiac magnetic resonance imaging [12], a technique which is often impractical in the postoperative period and is certainly not routine.

In a previous study of patients undergoing urgent orthopedic surgery, PMI, as defined by troponin I elevation, occurred in 52.9% of patients. The majority of biomarker elevations in these patients were asymptomatic, with only 9.8% of patients meeting the universal definition criteria for myocardial infarction. Whilst patients within each quartile of troponin elevation demonstrated increasing mortality at 12 months, mortality was dramatically increased across the board in those with any postoperative troponin elevation (37% versus 2.1%, *P* <0.0001) [13].

We subsequently performed an intervention study of patients with troponin elevation following emergent surgery for fractured neck of femur (NOF), and randomized patients to standard care or referral to the cardiology unit [14]. The main intervention by the cardiology unit was commencement of aspirin and beta-blockers, however this made no difference in mortality at follow-up. The study did confirm the high rate of troponin elevation in this group, and again confirmed the increased mortality conferred by postoperative troponin increase. It may be that efforts to prevent PMI will be more successful in improving outcomes following surgery rather than postoperative interventions directed at patients with PMI.

The exact mechanisms of PMI are not well understood, however two broad theories are postulated; myocardial stress and plaque rupture [15]. Prevention of myocardial stress and/or plaque rupture may reduce the incidence of PMI and therefore have been the subject of multiple studies.

### Myocardial stress (myocardial oxygen supply-demand imbalance) and role of ivabradine

Studies suggest that tachycardia is the most common cause of postoperative oxygen supply-demand imbalance leading to PMI [16, 17]. Numerous studies using perioperative Holter monitoring in patients undergoing major surgery have shown that asymptomatic, heart rate related ST segment depression is common postoperatively and associated with in-hospital [18, 19] and long-term morbidity and mortality [20]. Postoperative cardiac complications, including sudden death, occur after prolonged silent ST segment depression [11, 16, 21–25]. Furthermore, peak troponin elevation correlates with the duration of ST segment depression [16].

These studies are the basis for trials examining the use of perioperative beta-blockers to reduce the incidence of postoperative cardiac events. Initial small studies were promising [5, 26], however other moderate-sized randomized trials failed to show benefit [27, 28]. A subsequent meta-analysis of non-cardiac surgery randomized controlled trials did suggest a benefit for beta-blockers in the reduction of cardiovascular events, but showed an increased risk of hypotension and bradycardia [29]. The largest randomized controlled trial to date, the POISE trial (Effects of extended-release metoprolol succinate in patients undergoing non-cardiac surgery), randomized 8,351 patients at increased risk of cardiovascular events to receive metoprolol or placebo [30]. The group assigned to metoprolol showed a reduction in myocardial infarction (4.2 versus 5.7%, *P* = 0.002), however there was an increase rate of stroke (1.0 versus 0.5%, *P* = 0.005) and death (3.1 versus 2.3%, *P* = 0.03) in the treatment group. Clinically significant hypotension occurred in 625 patients (15%) in the metoprolol group. This higher incidence of clinically significant hypotension has been postulated as the cause for the occurrence of more strokes and deaths.

Postoperative atrial fibrillation (AF) is common both after cardiothoracic and non-cardiothoracic surgery [31]. In patients undergoing non-cardiothoracic surgery, the reported incidence of postoperative AF is as high as 12% [32], and has not been documented in an emergent orthopedic surgery group where the incidence may be higher given the typically elderly population. Postoperative AF can be observed throughout the postoperative course, with a peak on the second postoperative day [33]. Even though postoperative AF can be self-limiting, it may be associated with hemodynamic derangements, postoperative stroke, PMI, ventricular arrhythmias and heart failure [34, 35]. In many reports, development of postoperative AF is associated with a longer hospital stay, greater morbidity and mortality and increased costs [33, 36]. The pathophysiology of postoperative AF is not well understood, however right atrial overdistension and myocardial stretch in response to catecholamine release may play a significant role [37].

Ivabradine (Coralan™) is an orally bioavailable, specific inhibitor of the I_*f*_ current in the sinoatrial node [38]. It is a pure heart rate lowering agent in patients with sinus rhythm, but unlike beta-blockers it has no effect on blood pressure [39, 40], myocardial contractility [41], intracardiac conduction or ventricular repolarization [42]. Ivabradine could potentially provide the perioperative benefits of beta-blockers without the detrimental effects of hypotension, leading to improved outcomes and reduced adverse cardiovascular events in the postoperative period. A small series case study of 15 patients showed that ivabradine does attenuate catecholamine induced tachycardia following cardiac surgery, with positive effects on cardiac index, stroke volume index and mean arterial pressure [43]. A small (n = 111) non-randomized study of ivabradine in patients undergoing carotid endarterectomy found that ivabradine therapy (n = 33) reduced PMI compared to patients not receiving any beta-blockers or ivabradine [44]. No randomized studies with ivabradine in patients undergoing cardiac or non-cardiac surgery have been performed to date.

### Acute coronary syndromes and role of atorvastatin

An acute coronary syndrome occurs when an unstable or vulnerable plaque undergoes spontaneous rupture, fissuring or erosion, leading to acute coronary thrombosis, ischemia and infarction. Although it is accepted that intraplaque inflammation plays a pivotal role in plaque instability and spontaneous acute coronary syndrome [45], external stressors such as those occurring postoperatively are also believed to contribute [39, 40], especially in patients with vulnerable plaque characterized by a thin fibrous cap [18]. Indeed, the majority of perioperative troponin elevations occur in patients with underlying coronary artery disease (CAD) [10, 11, 46, 47]. Hormones that increase cardiovascular stress, such catecholamines and cortisol, increase after surgery and may remain elevated for days [48–50]. Elevation in plasma catecholamines correlates with postoperative cardiac troponin elevations [50] and with graft occlusion after vascular surgery [51]. Whilst myocardial oxygen supply-demand may be the predominant mechanism of injury, this remains unproven. The cardiovascular tension and shear stress induced by hemodynamic changes as a result of catecholamine increase may also induce plaque rupture or erosion leading to acute coronary thrombosis, ischemia and infarction. Increased platelet reactivity in the postoperative period may also be a significant contributor to plaque events leading to PMI [52].

Atorvastatin (40 mg orally daily) administered for seven days prior to percutaneous coronary intervention (PCI) reduces the incidence of PMI [53]. It has subsequently been shown that even a single high dose (80 mg) of atorvastatin within 24 hours prior to PCI significantly lowers the overall incidence of myocardial infarction not limited only to PMI rate [54].

A retrospective cohort study of 780,591 patients who underwent major non-cardiac surgery and who received statin therapy during the first two hospital days showed a reduction in crude and adjusted mortality in the statin group [55]. The authors concluded that ‘treatment with lipid-lowering agents may reduce risk of death following major non-cardiac surgery. Clinical trials are required to confirm this observation’. A subsequent review showed reduced death for statin therapy in cardiac and vascular surgery, but reported no randomized trials of statin therapy in non-cardiovascular surgery [56].

### Novel cardiac biomarkers

Elevated levels of circulating biomarkers related to cardiac volume, pressure overload and cardiovascular stress offer insights into subclinical cardiac stress and thus have the potential to aid in risk stratification and provide mechanistic data on disease prevention measures [57]. Specifically, B-type natriuretic peptide (BNP; either the hormone or the aminoterminal fragment of the prohormone (NT-proBNP)) is a counter-regulatory hormone released predominantly from ventricular myocytes in response to ventricular dysfunction, stretching, increased wall tension and ischemia. Elevated levels of BNP have been shown to predict mortality and heart failure events across a broad range of individuals, ranging from the general population to patients with coronary disease or heart failure [57–63]. We recently showed that elevated NT-proBNP levels pre- and postoperatively are independent predictors of in-hospital cardiac events and two-year mortality [64].

Development of newer assays that target more stable epitopes of hormones or prohormones that are released in relation to cardiomyocyte and/or vascular stress offers the potential for more refined risk assessment [65]. Specifically, atrial natriuretic peptide (ANP) is a vasodilator and natriuretic that is synthesized in the myocardium in response to increased wall tension [66]. Adrenomedullin (ADM) is a potent vasodilator synthesized in the adrenal medulla, vascular endothelial cells, heart and elsewhere in response to physical stretch and specific cytokines, with levels in the heart elevated in the setting of pressure and volume overload [67, 68]. Endothelin-1 (ET-1) is a potent vasoconstrictor and profibrotic hormone that is secreted by vascular endothelial cells, with levels correlating with shear stress and pulmonary artery pressure [69]. In patients with stable coronary artery disease and preserved left ventricular ejection fraction, elevated levels of these novel biomarkers can help identify patients who are at higher risk of cardiovascular death and heart failure [65]. Higher levels of these three biomarkers have been associated with an increased risk of death and/or heart failure events in patients with established heart failure [70–72].

The prohormones of ANP, ADM and ET-1 are released in equimolar ratios to the vasoactive hormones but have a longer half-life. The mid-regional (MR) fragments of proANP and proADM are also more stable *in vivo* and *ex vivo* than the amino- or carboxy-terminal part of the prohormone, thereby minimizing the risk of underestimation of levels as a result of early degradation of crucial epitopes at the extreme ends of the molecule [73]. In studies of patients with established heart failure, elevated levels of MR-proANP, MR-proADM and carboxy-terminal fragment of ET-1 (CT-proET-1) have been shown to be associated with mortality independently of clinical variables, and the biomarkers have displayed prognostic and discriminatory value that has compared favorably with BNP and/or NT-proBNP in the heart failure population [70–72].

#### Secretory Phospholipase A2

Secretory phospholipase A2 (sPLA2) and lipoprotein-associated phospholipase A2 (Lp-PLA2) are enzyme biomarkers of increased cardiovascular risk [74]. In addition, arachidonic acid release from membrane phospholipids by PLA2 is a key step in the control of eicosanoid. sPLA2 and Lp-PLA2 appear to be prospective candidates for inhibition [75]. High-dose atorvastatin significantly reduces sPLA2 and Lp-PLA2 mass and activity after acute coronary syndromes and mitigates the risk of death associated with sPLA2 mass. Atorvastatin may exert anti-inflammatory effects on phospholipases that contribute to its therapeutic benefit after acute coronary syndromes [74].

#### Lp-PLA2

Lp-PLA2 is a vascular specific marker that is not elevated in other inflammatory processes [76]. Lp-PLA2 is produced in atherosclerotic plaque by macrophages, T cells and mast cells, is highly concentrated in rupture prone lesions, and is bound to low-density and high-density lipoprotein (LDL and HDL) [77]. Statins have been shown to decrease Lp-PLA2 but not to the same extent as their effect on LDL levels, suggesting the potential for therapies that can more specifically target Lp-PLA2.

#### sPLA2

Type IIA sPLA2 is expressed in hepatocytes, macrophages, platelets and vascular smooth muscle cells, and its plasma levels are upregulated by proinflammatory compounds such as interleukin-1, interleukin-6, tumor necrosis factor, interferon and oxidized LDL. sPLA2 can also hydrolyze unmodified phospholipids, in contrast to Lp-PLA2 [78]. It has been shown that human atherosclerotic lesions contain extracellular and intracellular sPLA2 (macrophages and smooth muscle cells) [79]. sPLA2 is less vascular specific than Lp-PLA2. sPLA2 correlates with high sensitivity C Reactive Protein (hs-CRP) levels and is an acute-phase reactant [76]. Elevated sPLA2 levels predict coronary heart disease in patients with stable and unstable angina and normal subjects [80–84].

#### Assays

sPLA2 and Lp-PLA2 can be measured as mass or activity. There is some dispute over the best measurement to use. Most studies have used mass. There are a number of competing Lp-PLA2 mass assays on the market and different assays have been utilized in different studies with varying results reported. The United States Food and Drug Administration approved a blood test to measure Lp-PLA2 mass as a marker of cardiovascular disease (CVD) risk in 2005 (PLAC diaDexus, California, United States). Correlation between Lp-PLA2 mass and activity is variable depending on the clinical scenario as Lp-PLA2 levels are dependent on circulating LDL and HDL levels.

#### Coronary plaque burden

Underlying coronary disease is a prerequisite for acute coronary syndromes. Coronary artery calcification is a direct sign of atherosclerotic CAD [85, 86] and has been shown to be a strong predictor for risk of cardiovascular disease or events, including myocardial infarction and/or cardiac death [87–89]. The amount of coronary calcium can be quantified non-invasively by using computed tomography (CT) techniques and calculating the volume score [90]. The majority of perioperative troponin elevations occur in patients with underlying CAD [10, 11, 46, 47]. Conceivably, patients with a higher burden of CAD would derive the most benefit from interventions aimed at reducing PMI.

## Methods/Design

This is a prospective, single-centre (Northern Hospital, Victoria, Australia), open-label, 2x2 factorial, randomized controlled trial of ivabradine and atorvastatin in the prevention of myocardial injury following emergent orthopedic surgery for lower limb fracture. The Northern Hospital Ethics Committee approved this study on 6 July 2012 (reference number HREC P06/12). This study complies with the Declaration of Helsinki. Informed consent will be obtained from all participating patients. Upon signing informed consent, the patient’s data will be populated as per protocol.

### Primary outcome measure

Based on the peak troponin I level postoperative day 1, 2, 3 and 4, the primary outcome measure will be the frequency (binary outcome) and magnitude (continuous variable) of new myocardial injury following emergent orthopedic surgery for lower limb fracture. A troponin I level of less than 0.04 μg/L is considered positive in our reference laboratory.

### Secondary outcome measures

The secondary outcome measures will be as follows: myocardial infarction according to the universal definition [8]; death in-hospital, at 30 days and 12 months; stroke in-hospital, at 30 days and 12 months; markers of myocardial stress: NT-proBNP (pmol/L), MR-proANP (pmol/L), MR-proADM (nmol/L) and CT-proET-1 (pmol/L); and markers of plaque burden: sPLA2 and Lp-PLA2 mass (%) and activity (%). There are also several outcomes that will be monitored for safety purposes: symptomatic bradycardia or heart block requiring the cessation of ivabradine, and liver enzyme elevation more than three times the upper limits of normal (ALP, GGT, AST and ALT measured in U/L) requiring the cessation of atorvastatin.

### Study population

The study aims to recruit 200 patients over the age of 60 years admitted to Northern Hospital with fractured neck of femur who are planned to undergo surgery within 48 hours.

### Inclusion criteria

Patients may be included in the trial if they are over 60 years of age and have a NOF fracture with surgical treatment planned for within the next 48 hours.

### Exclusion criteria

Patients who meet any of the following criteria will be excluded from the study: 1) current ivabradine use, current atorvastatin use of 80 mg daily, 3) heart rate <65 bpm prior to randomization, 4) known liver disease or liver enzymes more than three times the higher limit of normal, 5) preoperative troponin elevation, 6) artificial pacemakers, sick sinus syndrome or complete heart block, 7) concomitant CYP3A4 inhibitors (ketoconazole, macrolides, cyclosporin, gestodene and antiretrovirals), 8) significant cognitive disorders, and 9) patients who do not undergo surgical treatment for lower limb fracture for any reason. If such patients were initially enrolled they will be excluded from the analysis (per protocol analysis).

### Randomization protocol

Patients will be randomized using a computerized block randomization method with blocks of variable sizes to ensure ‘true’ randomization and equal numbers in each treatment group. Patients will be randomized to one of four groups in a 1:1:1:1 ratio: no treatment (control group), atorvastatin 80 mg daily, ivabradine twice daily (as per protocol), or ivabradine twice daily (as per protocol) and atorvastatin 80 mg daily.

### Ivabradine protocol

Open-label ivabradine will be administered according to the following protocol.

Heart rate 65 to 90 bpm at randomization: starting dose of 5.0 mg administered orally twice daily commencing immediately after randomization (within two hours and prior to surgery) and continued until discharge. Heart rate >90 bpm at randomization: starting dose of 7.5 mg administered orally twice daily commencing immediately after randomization (within two hours and prior to surgery) and continued until discharge. ivabradine dose and heart rate will be reviewed on a daily basis by the cardiology research fellow (associate investigator) and adjusted as follows: 1) if heart rate remains over 65 bpm during treatment then ivabradine dosage will be increased by 2.5 mg twice daily to a maximum of 7.5 mg twice daily; 2) if heart rate falls below 50 bpm during treatment then ivabradine dosage will be reduced by 2.5 mg twice daily; and finally 3) if heart rate remains below 50 bpm on ivabradine 2.5 mg twice daily then ivabradine will be ceased.

### Atorvastatin protocol

Open-label atorvastatin will be administered according to the following protocol. A dosage of 80 mg administered orally daily will commence immediately after randomization (within two hours and prior to surgery) and will continue until discharge (unless otherwise indicated according to history and lipid profile). A liver function test and creatinine kinase levels will be monitored to assess side effects.

### Electrocardiography protocol

A digital 12-lead ECG will be recorded preoperatively and on postoperative days 1, 2 and 3 and assessed for heart rate, PR interval, QRS duration and morphology, Q waves, QT duration and QTc, as well as ST-T morphology and overall interpretation to look for changes that meet the universal definition of myocardial infarction.

### Echocardiography protocol

A transthoracic echocardiogram will be done on every patient prior to discharge to assess for evidence of regional wall motion abnormality, systolic and diastolic dysfunction. Images of the left ventricle in motion and hemodynamic measures will be recorded at rest.

### Holter protocol

Continuous 48-hour Holter monitoring will be commenced within 24 hour postoperatively to assess for heart rate, postoperative arrhythmia including atrial fibrillation, its frequency and its duration.

### CT coronary calcium protocol

A non-invasive, non-contrast CT coronary calcium score will be acquired on a 320-detector row CT scanner (Toshiba Aquilion ONE, Toshiba Medical Systems, Otawara, Japan) prior to or at discharge following surgery. The scan range will be planned between the carina and cardiac apex. Depending on the expected scan range, a 320 × 0.5 mm or a 280 × 0.5 mm detector configuration will be used as previously described [91]. Immediately before image acquisition, an optimal reconstruction phase will be automatically determined during a breath hold exercise with ECG-recording by use of cardiac scanning software (SureCardio, Toshiba Medical Systems, Otawara, Japan). Full cardiac calcium score acquisition will be performed in a single gantry rotation (0.35 seconds) during breath hold at inspiration that allows image reconstruction at a single cardiac phase. Scan parameters: tube voltage 120 kV and tube current 200 to 400 mA (dependent on patient size and shape as visually assessed by the radiology technician: 200 mA for small and/or thin patients, 250 mA for normal patients and 300 to 400 mA for large and/or obese patients).

Image reconstruction will be performed using a standard reconstruction kernel filter from the cardiac scanning software (SureCardio, Toshiba Medical Systems, Otawara, Japan) with a 200 to 220 mm^2^ field of view. Non-overlapping 0.5 mm datasets will be reconstructed for evaluation of coronary calcium, and the reconstructions transferred to a post-processing workstation for analysis.

Calcium volume scores (Table 1) will be performed on a post-processing workstation (Vitrea FX, version 1.0, Vital Images, Minnetonka, United States), using calcium score analysis software (VScore, Vital Images). Coronary calcium will be defined as an area of at least three ‘face-connected’ voxels in the axial plane in the course of a coronary artery, with an attenuation threshold-value of ≥130 Hounsfield units (HU).

**Table 1:** **Calcium score interpretation**

Translation of calcium score		
Calcium score	Implication	Risk of coronary artery disease
0	No identifiable plaque	Very low, generally less than 5%
1 – 10	Minimal identifiable plaque	Very unlikely, less than 10%
11 – 100	Definite, at least mild atherosclerotic plaque	Mild or minimal coronary narrowings likely
101 – 400	Definite, at least moderate atherosclerotic plaque	Mild coronary artery disease highly likely, significant narrowings possible
401 or higher	Extensive atherosclerotic plaque	High likelihood of at least one significant coronary narrowing

### Troponin I

Serum for troponin I measurement will be taken preoperatively (within two hours of randomization) and on days 1, 2, 3 and 4 postoperatively. Serum will be stored at -70°C. Prior to analysis, the frozen serum will be thawed, thoroughly mixed and re-centrifuged.

Troponin I will be measured using the Architect STAT Troponin I assay (Abbott Diagnostics, Abbot Park, IL, USA). The 99th percentile of a healthy reference population using this assay has been previously established at 0.03 mcg/l and levels above this are considered indicative of myocardial injury. The coefficient of variation (CV) for this assay is 10% at the 99th percentile.

### Markers of cardiovascular stress

Levels of MR-proANP, MR-proADM and CT-proET-1 will be determined using the Time-Resolved-Amplified-Cryptate-Emission (TRACE) technology on the Kryptor Compact analyzers (BRAHMS GmbH, Henningsdorf, Germany), from samples collected at the same time points as troponin.

T-pro-BNP levels will be measured using the Elecsys electrochemiluminescence immunoassay (ECLIA) proBNP II assay on a Roche Cobas E170 analyzer (Roche, Indianapolis, IN), from samples collected at the same time points as troponin. This assay uses two polyclonal antibodies that recognize epitopes located on the N-terminal part of pro-BNP. The lower limit of the detection range (sensitivity) is 5 pg/ml. Within-run coefficients of variation are 1.9% at a concentration of 64 pg/ml and 1.3% at 14,142 pg/ml. Total precision coefficients of variation are 3.1% at a concentration of 36 pg/ml and 2.7% at 125 pg/ml. Levels of the biomarkers of the renin-angiotensin pathway, including Ace2, will be measured.

### Markers of plaque burden

Total cholesterol, triglycerides, HDL and LDL will be measured at randomization (fasting sample just prior to surgery) and again on day 2 postoperatively (fasting sample), using the same venipuncture as the preoperative and day 2 troponin measurement.

sPLA2 and Lp-PLA2 mass and activity assays will be performed at the same time point as fasting lipids. Lp-PLA2 and sPLA2 mass will be measured with an ELISA based assay (sPLA2: Cayman Chemical Co., Ann Arbor, Michigan, United States and Lp-PLA2: diaDexus Co. California, United States). sPLA2 activity is measured by a fluorometric assay. All blood samples will be collected and put on ice prior to centrifugation, separation and storage at –80°C in a dedicated secure freezer. Batch testing will then be performed at a later date.

### Follow-up protocol

All patients will be followed up on by means of a semi-structured interview (via telephone call) at 30 days, 6 months and 1 year, enquiring about any cardiac events, hospitalizations, functional level and medication usage. Additionally their medical records will be retrieved and/or primary physician contacted for more information. Deaths will be confirmed with the Births, Marriages and Deaths registry.

### Sample size calculation

We estimated that 200 patients (calculated sample size of 171) would be required to have a power of 0.90 (alpha = 0.05, two tail) to identify a difference(moderate effect size (f = 0.25) in PMI incidence between the 4 groups. (F test Anova:fixed effects,special,main effects and interactions; Analysis: a priori).

We plan to recruit 200 patients in total (50 in each treatment cell), providing an actual power of 0.94 to detect a moderate effect size.

### Statistical analysis

A comprehensive statistical analysis will be performed using SPSS (IBM, USA) for the *a priori* hypotheses. Baseline characteristics will be reported as mean ± SD for normally distributed continuous variables, and as counts (percentages) for categorical variables. The Spearman correlation will be used to calculate the association between different biomarkers. A Cox proportional-hazards model will be used to examine the association between biomarker levels and outcomes. A value of *P* <0.05 will be considered statistically significant.

## Discussion

Through this study, we postulate that ivabradine will reduce the rate and magnitude of PMI following emergent lower limb orthopedic surgery by reducing heart rate and attenuating catecholamine-induced tachycardia postoperatively. Secondly, we postulate that postoperative reduction in heart rate and catecholamine-induced tachycardia with ivabradine will correlate with a reduction in biomarkers of cardiovascular stress as measured by NT-proBNP, MR-proANP, MR-proADM and CT-proET-1, which will reduce atrial stretch and postoperative incidence of atrial fibrillation. We further postulate that atorvastatin will reduce the rate and magnitude of PMI following emergent lower limb orthopedic surgery via a reduction in sPLA2 and Lp-PLA2 mass.

To conclude, we aim to demonstrate that treatment with ivabradine and atorvastatin will cause a reduction in the incidence and magnitude of PMI, the benefit of which is derived primarily in patients with greater atherosclerotic burden as measured by higher CT coronary calcium scores.

## Trial status

This study is currently recruiting participants since 2012 and finishes once 200 patients are recruited.

## Abbreviations

Ace2: Angiotensin-converting enzyme 2
ADM: Adrenomedullin
ADP: Adenosine diphosphate
AF: Atrial fibrillation
ANP: Atrial natriuretic peptide
BNP: B-type natriuretic peptide
CAD: Coronary artery disease
CT: Computed tomography
CT-proET-1: Carboxy terminal fragment of ET-1
ECG: Electrocardiogram
ECLIA: Elecysys electrochemiluminescence immunoassay
ENOS: Endothelial nitric oxide synthase
ET-1: Endothelin-1
HDL: High-density lipoprotein
hs-CRP: High sensitivity C-reactive protein
HU: Hounsfield units
LDL: Low-density lipoprotein
Lp-PLA2: Lipoprotein-associated phospholipase A2
MR: Mid-regional fragments
NOF: Neck of femur
NT-proBNP: Aminoterminal fragment of prohormone B-type natriuretic peptide
PCI: Percutaneous coronary intervention
PMI: Perioperative myocardial infarction
sPLA2: Secretory phospholipase A2
TRACE: Time-resolved amplified cryptate emission.
